# Prevalence of risk for dyslexia, risk for dyscalculia, and their comorbidity in Spanish primary education: gender difference and socioeconomic status

**DOI:** 10.3389/fpsyg.2025.1664437

**Published:** 2025-10-28

**Authors:** Mariana Loredo, Juan L. Luque, Almudena Giménez, P. Javier López-Pérez

**Affiliations:** ^1^Neuroscience and Education Laboratory, Leeduca, Department of Developmental and Educational Psychology, University of Málaga, Málaga, Spain; ^2^Developmental Psychology and Psychopathology Group, Faculty of Psychology, Universidad de la Costa, Barranquilla, Colombia

**Keywords:** dyslexia, dyscalculia, comorbidity, socioeconomic status, gender, prevalence, specific learning disorders

## Abstract

Dyslexia and dyscalculia frequently co-occur; however, population-based estimates from Spain, remain scarce. This study aims to assess the prevalence of risk for dyslexia (RDyx), risk for dyscalculia (RDC), and their comorbidity (RDyx+RDC) and to evaluate their distributions by gender and socioeconomic status. A total of 691 Spanish students in 5th–6th grade were assessed with computerized screening tasks in reading and mathematics. Risk groups were defined using a cut-off of −1 SD (16th percentile) within each domain. Prevalence was 8.5% (6.6–10.9%) for RDyx, 4.2% (2.8–6.0%) for RDC, and 2.0% (1.1–3.4%) for RDyx+RDC. Comorbidity exceeded chance expectations and was asymmetrical: 48.3% of children with RDC also presented reading difficulties, while 23.7% of children with RDyx showed concurrent math difficulties. Gender effects were significant for RDC, with girls showing higher odds than boys (OR = 3.16), whereas no significant gender effect was observed for RDyx (OR = 1.56). Socioeconomic status showed a marginal effect in RDyx, but no effects were observed for RDC or comorbidity. These results provide the first population-based prevalence estimates of RDyx, RDC, and their comorbidity in Spanish primary education and underscore the value of risk-based digital screening at the school level.

## Introduction

Recent theoretical frameworks have emphasized that learning disorders (LDs) should not be regarded as discrete categories but rather as dimensional continua in which clinical and subclinical manifestations coexist (Carroll et al., 2025; Catts and Petscher, 2022; Pennington, 2006). In line with this perspective, the recent international Delphi consensus on dyslexia highlighted that reading difficulties exist on a continuum of severity and frequently co-occur with other developmental disorders (Carroll et al., 2025). Research also shows that children who exhibit difficulties in learning to read are likely to manifest other learning difficulties. One of the LDs that more frequently cooccur with dyslexia is dyscalculia, the difficulty to acquire arithmetic skills (Koponen et al., 2018; Moll et al., 2020; Pedemonte et al., 2024; van Bergen et al., 2025). The overlap between dyslexia and arithmetic deficits is reported to be 2–3 times higher than would be expected by chance (Kaufmann and von Aster, 2012; Landerl and Moll, 2010). In addition to neurocognitive vulnerabilities, sociodemographic factors such as gender and socioeconomic background play a crucial role in shaping the expression and detection of learning difficulties (Buckingham et al., 2014; Girard et al., 2022). Previous research indicates that the probability of learning disabilities differs by gender (Arnett et al., 2017; Francés et al., 2023; Quinn and Wagner, 2015). Boys have been found more likely than girls to experience reading difficulties (Quinn and Wagner, 2015; Rutter et al., 2004). In contrast, results respect to dyscalculia are mixed (eg. Barbaresi et al., 2005; Devine et al., 2013; Morsanyi et al., 2018; Poltz et al., 2025). Furthermore, disorders and their comorbidity should be understood as reflecting overlapping cognitive and environmental risk factors that may interact to varying degrees across individuals (Catts and Petscher, 2022). Reading and maths achievement is mediated by the frequency and quality of the provision of educational inputs (Aikens and Barbarin, 2008). Children from economically deprived families have less supportive parents, are exposed to less rich language, participate in more impoverished interactions, and may attend to less well-equipped schools than do children from higher socioeconomic status (SES) families (Neuman et al., 2018). Under these conditions, children from low SES families are at a disadvantage to cope with schooling and likely to be at risk. Beyond indicating the frequency with which LDs prevail differently across gender and SES, prevalence data can provide insights into how these factors interact to exert a promotive or protective effect. In Spanish, the available prevalence studies on comorbidity between dyslexia and dyscalculia are scarce and fail to pay attention to SES and gender variables (Bosch et al., 2021; Carballal et al., 2018; Francés et al., 2023; Villegas, 2023). Therefore, in line with recent consensus that specific learning disorders should be conceptualized as dimensional and frequently comorbid phenomena (Carroll et al., 2025; Catts and Petscher, 2022), the present study pursued two main aims. First, we estimated the prevalence of risk for dyslexia (RDyx), risk for dyscalculia (RDC), and their co-occurrence in a large population-based sample of Spanish 5th- and 6th-grade students, also examining differences by gender and socioeconomic status (SES). Second, rather than relying on rigid diagnostic cut-offs, we adopted a risk-based approach anchored in the 16th percentile criterion, which better reflects the continuum of difficulties and their interaction with sociodemographic factors. By applying computerized, school-based screening tools, this work contributes to the development of universal identification strategies that can inform timely and cost-effective educational interventions.

### Dyslexia

The recent international Delphi consensus reached a broad agreement on how dyslexia should be conceptualized. The expert panel concluded that dyslexia is best understood as a constellation of processing difficulties that primarily affect the acquisition of reading and spelling, despite adequate educational opportunities. These difficulties are characterized by persistent problems in reading fluency and spelling, and they exist along a continuum of severity rather than constituting a discrete category. Importantly, the Delphi consensus also emphasized that dyslexia frequently co-occurs with other developmental conditions, including dyscalculia, developmental language disorder, ADHD, and developmental coordination disorder (Carroll et al., 2025). Moreover, the panel highlighted that the manifestation of dyslexic symptoms may change as literacy skills are acquired and consolidated: whereas younger children often show marked decoding problems, older students may primarily experience persistent difficulties in fluency, spelling, or higher-level comprehension (Shaywitz et al., 1999; Snowling et al., 2007; Snowling et al., 2020). This developmental perspective converges with the cumulative risk–resilience model of dyslexia (Catts and Petscher, 2022), which situates reading difficulties as the outcome of interacting vulnerabilities and protective factors rather than of a single underlying deficit.

Dyslexia has been extensively investigated given its profound academic and personal consequences, yet estimated prevalence shows striking variability across studies. In opaque orthographies, reported rates range widely from 3.9 to 20%, reflecting differences in populations and methodological choices (Dirks et al., 2008; Di Folco et al., 2021; Katusic et al., 2001; Lindgren et al., 1985; Shaywitz et al., 2021; Lewis et al., 1994). Spanish-speaking contexts also illustrate this variability. A recent meta-analysis estimated that approximately 7.5% of school-aged children meet diagnostic criteria for dyslexia (Cubilla-Bonnetier, 2024). However, individual studies report diverse figures: Cuadro et al. (2017) found rates between 2.2 and 5.3% in a large school-based sample from Uruguay, Cubilla-Bonnetier et al. (2021) documented 5.56% in Panamanian students from 4th to 6th grade, and Spanish studies using standardized instruments have reported prevalence ranging from 3.2 to 10.9% (Jiménez et al., 2009; Carrillo et al., 2011).

Moreover, epidemiological studies conducted in Spain continue to reveal substantial heterogeneity. Early investigations already reported prevalence estimates between 2.0 and 15.2% (Carballal et al., 2018; Carrillo et al., 2011; Jiménez et al., 2009). More recent large-scale studies confirm this lack of convergence: Francés et al. (2023) identified 9% of six-year-olds with reading difficulties in a population-based cohort, Bosch et al. (2021) reported 9.28% in a large school sample, while Villegas (2023) found prevalence rates of 1.24% under a stringent cutoff and 3.6% under a more lenient criterion in a cohort of nearly one million students. Collectively, these findings illustrate that prevalence estimates of dyslexia vary greatly and are highly sensitive to methodological decisions—including diagnostic criteria, assessment instruments, and cutoff thresholds—making direct comparisons across studies challenging.

The wide variability reported in the reviewed studies highlights the limitations of categorical approaches to capturing learning disorders. Therefore, it is advisable to develop instruments that provide objective screening of risk levels, which can then be complemented by other cognitive, sociodemographic, and protective factors to guide educational decision-making and, when appropriate, to inform clinical diagnosis.

### Dyscalculia

Dyscalculia is defined as a specific impairment affecting the acquisition of numerical and arithmetic skills, including difficulties in number sense, calculation, and arithmetic fact retrieval (Butterworth et al., 2011; Geary et al., 2007; Piazza et al., 2010; von Aster and Shalev, 2007). Despite previous research demonstrates that dyscalculia is present across diverse populations it has received considerably less research attention than dyslexia (Han, 2025; Shalev, 2004).

Epidemiological evidence reported comparable rates from diverse populations (Espina et al., 2022). A twin study including more than 19,000 children from the Netherlands obtained a prevalence of 10.2% (van Bergen et al., 2025). In Italy, Luoni et al. (2023) documented a prevalence of 9.7% in a cohort of 8- to 9-year-olds. In Ireland, Morsanyi et al. (2018) found that 5.7% of primary school children displayed dyscalculia. Differences in cutoff thresholds can partly account for rate variation across studies (Devine et al., 2013). As can be observed in Table 1, the percentage of children detected doubles when a lenient criterion is used. To take just a pair of examples, Moll et al. (2014) reported a prevalence of 4.9 with a criterion of −1.5 standard deviation (SD), but prevalence increased to 12.9 when the limit to be included in the dyscalculia group was −1 SD. Similarly, in Southeast Brazil, Santos et al. (2022) reported prevalence rates ranging from 4.6 to 7.4%, depending whether the criterion applied was fifth percentile or −1.5 SD, respectively.

**Table 1:** Prevalence studies on comorbidity between dyslexia and dyscalculia.

Study	Country	*N*	Age	Cutoff	DC	Dyx	Comorbid	DC of Dyx	Dyx of DC
Lewis et al. (1994)	UK	1,056	9–10	<16th pc	1.3	3.9	2.3	37	64
Gross-Tsur et al. (1996)	Israel	3,029	11–12	20th pc + 2 years delay	6.5	-	0.8	-	17
Badian (1999)	USA	1,075	7–8	<25th pc	2.3	6.6	3.4	-	-
			6–14	<20th pc	6.9	9	3.0	-	-
Koumoula et al. (2004)	Greece	240	7–11	< −1.5 SD	6.3	8.4	2.1	-	-
von Aster et al. (2007)	Switzerland	337	6–8	−1.5 SD	1.8	3.3	4.2	56	70
Dirks et al. (2008)	Netherlands	799	8–12	<25th pc	10.3	19.9	7.6	28	43
				<10th pc	5.6	8	1	11	15
Landerl and Moll (2010)	Austria	2,586	7–10	−1 SD	15.4	14.8	5.7	37.3	38.8
				−1.5 SD	6.1	7.0	1.5	25.9	22.7
Moll et al. (2014)	Germany	1,633	8–10	−1 SD	12.9	15.5	1.84	34.3	41
				−1.25 SD	7.7	9.9	0.6	25.2	32.2
				−1.5 SD	4.9	7.1	0.4	21.6	30.2
Morsanyi et al. (2018)	Ireland	2,421	7–11	−1.5 SD	5.7	4.4	0.3	-	5.6
Luoni et al. (2023)	Italy	380	8–9	≤ − 2 SD math; < 5th pc RA or < −1.5 SD RF	9.7	-	6.1	-	62.2
Aro et al. (2023)	Finland	2,140	7–13	1.5 SD	11.5	12.3	7.1	-	-
Poltz et al. (2025)	Germany	1,264	7–12	−1.5 SD	6.0	7.4	0.3	-	-
van Bergen et al. (2025)	Netherlands	21,275	7–10	10th pc	10.2	9.8	1.9	-	-

SD, standard deviation; pc, percentile; −, information not specified in the article; DC, dyscalculia; Dyx, dyslexia; Comorbid, children with both disorders; RA, reading accuracy; RF, reading fluency.

To our knowledge, only two studies examined the prevalence of dyscalulia among Spanish-speaking populations. Francés et al. (2023) identified mathematical learning difficulties in 3.11% of 6-year-old Spanish children. A comparable estimation of 3.4% was reported by Reigosa-Crespo et al. (2012) in a sample of Cuban children when applying a cutoff of −2 SD in both a mental arithmetic task and one additional screening task, whereas the proportion increased to 9.35% when the cutoff was applied to the mental arithmetic task alone.

### Comorbidity

Accumulated findings highlight that the co-occurrence between reading and arithmetic difficulties is consistently high across studies, (Landerl et al., 2013; Moll et al., 2020; Pedemonte et al., 2024; van Bergen et al., 2025). In a study with a sample of 2586 children, Landerl and Moll (2010) found that showing a core deficit in reading or in numerical processing makes a child 4 to 5 times a more likely candidate to present both deficits. More recently, Joyner and Wagner (2019) and van Bergen et al. (2025) calculated that the probability of meeting the criteria for dyscalculia doubled when dyslexia was already present. Despite some individuals have only a single deficit, the high degree of comorbidity shown by existing research suggests that the profile of children with LDs are better characterized by a combination of deficits that differ in degree and distribution (Joyner and Wagner, 2019).

A relevant finding is that comorbidity may not be symmetrical. With a few exceptions (e.g., Landerl and Moll, 2010, see Table 1), children with dyscalculia exhibit dyslexia in a higher proportion than children with dyslexia who show concurrent dyscalculia. One possible explanation for the co-occurrence between math and reading disabilities is their shared reliance on language skills (Hecht et al., 2001; De Smedt et al., 2010). Number names, multiplication rules or the retrieval and communication of numeral facts are supported on a linguistic (phonological) code (Dehaene, 1997). As a result, children with dyscalculia may be more likely to experience reading difficulties due to the phonological requirements inherent in mathematical tasks (Yang et al., 2022). In contrast, children with dyslexia may present more domain-specific impairments that do not necessarily interfere with numerical reasoning to the same extent.

Taking together, these findings emphasize the need to conceptualize dyslexia and dyscalculia as potentially overlapping profiles rather than as isolated conditions. Despite previous evidence on the comorbidity between dyslexia and dyscalculia, no large-scale prevalence study has yet examined this issue within the Spanish population using a systematic screening procedure. It is essential to know the prevalence of these disorders, both in isolation and in their comorbid occurrence. This approach may have crucial consequences to establish early identification protocols and design interventions tailored to the complexity of learning profiles in educational settings (Starling-Alves et al., 2025).

### The role of gender and socioeconomic status

Beyond cognitive risk factors, sociodemographic variables may play a crucial role in shaping the prevalence and expression of LDs. Among these, gender has been widely studied in relation to dyslexia. Large-scale investigations have consistently reported a higher prevalence in boys, with gender ratios ranging from 1.6:1 to 2.8:1 (e.g., Rutter et al., 2004; Quinn and Wagner, 2015). These differences may reflect a male vulnerability to developing dyslexia. However, other factors, such as referral biases in educational settings or the diagnostic criteria used to define the disorder, may also influence (Liederman et al., 2005; Shaywitz et al., 1990; Siegel and Smythe, 2005).

Respect to the literature on gender differences in dyscalculia, some studies find no significant gender-based variation (Devine et al., 2013; Gross-Tsur et al., 1996; Lewis et al., 1994), while others report a slight overrepresentation of girls (Dirks et al., 2008; Luoni et al., 2023; Moll et al., 2014; Poltz et al., 2025).

Such asymmetry may be influence by social and economic factors rather than reflecting a biological predisposition. In the case of the overrepresentation of boys in dyslexia, Shaywitz et al. (1990) argue that it can be attributed to their tendency to exhibit more overtly disruptive and attention-seeking behaviors. Such behaviors are more likely to draw the attention of teachers, leading to a higher rate of referrals for clinical evaluation among boys compared to girls. In the case of dyscalculia, anxiety, gender stereotypes, or imposed expectations may partly account for the disproportion in diagnoses (Goetz et al., 2013; Hyde and Mertz, 2009; Rossi et al., 2022).

Given its strong impact on academic outcomes and its influence as a risk factor for the development of dyslexia and dyscalculia, a relevant variable to consider is the families socioeconomic status (SES) (Catts and Petscher, 2022). Children from low-SES backgrounds are disproportionately affected by these disorders due to a combination of environmental and psychosocial risk influences. Some of these factors are cognitive stimulation, greater exposure to stress, and reduced access to educational resources (Fernald et al., 2013; Tan, 2024). In the domain of literacy, previous studies indicate that children from disadvantaged families are more likely to experience reduced exposure to rich language environments and fewer early literacy experiences, such as shared book reading or access to age-appropriate books (Hoff, 2003; Raikes et al., 2006). These limitations hinder their opportunities for vocabulary acquisition, phonological awareness and comprehension, all of which are essential for developing proficient literacy skills (Buckingham et al., 2014; Hoff, 2003).

In mathematics, evidence indicates that children from low-SES households often enter school with delays in early numerical competencies (Jordan et al., 2009). This may be attributed to differences in the quality and frequency of math-related activities and resources provided by parents at home (Dunst et al., 2017; Elliott and Bachman, 2018). Indeed, children who engage more regularly in home numeracy practices tend to demonstrate stronger mathematical abilities than their peers (Elliott and Bachman, 2018; Levine et al., 2010). Other studies have found that SES influences the development of numerical skills and it is associated to the likelihood of presenting dyscalculia (Jordan et al., 2009; Li et al., 2025).

Given this evidence, it could be argued that SES plays a fundamental role in the development of skills for reading acquisition. Therefore, it is essential to conduct population-based studies in diverse sociocultural settings to obtain representative prevalence estimates across different population subgroups.

### The present study

New insights of reading disability assume that identification and risk prediction is not such a simple goal for various reasons. Research examining these deficits at the individual level reports that learning disabilities frequently co-occur (Pennington, 2006; Moll et al., 2020). Of particular interest for this study, children who exhibit difficulties in learning to read often manifest arithmetic difficulties (Carroll et al., 2025; Landerl and Moll, 2010). According to this view, once a child is identified as at risk of one of these disorders, they become a likely candidate for developing the other disorder.

Furthermore, it has been recognized that factors such as the social interactions and opportunities experienced by the child may also influence the achievement of reading or arithmetic skills (van Bergen et al., 2016). Children who grow up in enriched environments have more opportunities to receive stimulation or participate in activities that can contribute to compensate for their difficulties. In contrast, if the family has limited resources, children are more likely to be less prepared to cope with the demands of school, which can lead to a worsening of their difficulties or delay their learning outcomes. In the same line, most studies report unequal prevalence rates of reading and arithmetic disorders in boys than girls. However, results on gender influence are mixed and need further investigation. This led us to consider the potential role of gender and SES. Furthermore, the inclusion of gender and SES lends the opportunity to examine the complex relationship between the factors involved in the occurrence of learning difficulties (Catts and Petscher, 2022).

A further problem is that developmental learning disabilities (e.g., dyslexia and dyscalculia) manifest in a continuous rather than a categorical fashion (Shaywitz et al., 2008), ranging from relatively mild (even subclinical) difficulties to severe impairments (Hulme and Snowling, 2009). However, when considering whether a child experiencing difficulties, it is necessary to use a cut-off criterion, even if this means that the established cut-off point will determine the number of children identified. If it is very restrictive, some children, whose difficulties are not severe enough to pass the criteria, will fall out of receiving specific attention. On the contrary, a lenient cut-off is likely to include children who do not require attention (Snowling and Melby-Lervåg, 2016). In this study, 1 standard deviation was the cut-off criterion to identify reading or arithmetic difficulties, a point that represented the 16th percentile in the distribution produced by our sample. Given the impact of the cutoff on diagnosis and subsequent intervention, it was appropriate to use a limit that would capture individuals who will not reach more stringent criteria for but still experience significant problems compared to their peers. In addition, this criterion is commonly used, especially in screening or risk identification studies, where even less stringent limits are used (Snowling and Melby-Lervåg, 2016) what allows us to compare our results with previous studies and, at the same time, is in line with the objectives of a screening tool.

Then, the co-occurrence of reading and arithmetic impairments, the continuous nature of reading, and the contribution of demographic factors seem to suggest a comprehensive approach to identify children at risk of learning disabilities (Shaywitz et al., 2008).

Finally, the interest in identifying prevalence rates for reading and arithmetic disorders and its distribution across demographic variables lies in its utility for education practitioners. The probability that a specific disorder exists in a given population could help education practitioners for taking intervention decisions to prevent or limit the extent of a child’s difficulties. Thus, results could provide a link between theory and practice.

Risk identification relies on educational assessments, such as those employed in this study, to detect students whose reading or mathematical performance falls below expected levels. However, it is important to note that dyslexia and dyscalculia are clinical diagnoses that require comprehensive evaluation by specialists, including standardized test scores (Mather and Wendling, 2024; Shalev and Gross-Tsur, 2001). Identifying students at risk for these conditions is a first step in the diagnosis and intervention process.

The present study was carried out to further investigate the usefulness of a set of tasks for the identification of risk for dyslexia (RDyx), risk for dyscalculia (RDC). As a distinctive feature, the tasks used here were designed to be administered by the school staff in the school context as part of the school ordinary activities. Consistent with it, tasks should comply with the restrictions of space, time, and staff availability of the school to be administered effectively. The assessment is based on a few selected short tasks that could be self-administered under the guidance of even non-qualified education practitioners (teachers, assistants) who receive brief training. Children access tasks and data are collected via an online platform using computers or tablets in regular classes. These measures were combined with information on gender and SES.

Importantly, in this study a large population-based sample of school children at the end of primary participated. This allowed us to recruit children who fell at all points along reading and arithmetic achievement continua and distributed across the full range of SES. These characteristics made the assessed population especially suitable to obtain unbiased information on comorbidity ratios between dyslexia and dyscalculia. In addition, it may be informative with respect to the role of gender and SES in the distribution of the disorders under study.

The specific research questions were as follows:

Research Question 1: What are the prevalence rates of RDyx, RDC, and their comorbidity among Spanish primary school students? It is expected to find similar ratio limits to prior research.

Research Question 2: Are there significant gender differences in the prevalence of RDyx and RDC? Based on previous research, it is expected to find unbalance prevalence ratios related to gender.

Research Question 3: Does SES influence the prevalence of RDyx and RDC, whether isolated or co-occurring? As commented above, many studies have reported higher vulnerability for learning problems in children coming from socioeconomic deprived environments. According to this view, children from low SES will be more likely expected to manifest reading or arithmetic problems.

Research Question 4: Are short, computerized tools appropriate for screening aims? A relevant objective is to test the effectiveness of the procedure used in order to extending it to other populations and earlier levels of education. If similar prevalence ratios are obtained, the method can be considered adequate.

## Method

### Participants

A total of 812 students attending 5th and 6th grade coming from 17 public schools in urban and suburban areas distributed all over the province of Málaga, Spain to obtain a representative sample. The last two grades of primary education were chosen because at this age most children have acquired the basic reading and mathematics skills, and less time-based changes are expected (Roman et al., 2009). Private schools, situated in high SES urban areas and mostly associated to religious communities, were discarded to reduce the biases due to school idiosyncrasies. 105 children who did not complete the full battery, and 16 identified as non-Spanish speakers were excluded from analyses. The final sample consisted of 691 students (352 girls, 339 boys) with mean age 11.4 years (SD = 0.5). The SES was estimated using the SES index assigned to each school, a proxy measure frequently employed in the Spanish educational system to represent the socioeconomic background of the students. Based on this index, 33.7% of participants attended schools classified as low SES (*N* = 233), 38.7% as average SES (*N* = 268), and 27.5% as high SES (*N* = 190) (see Table 2).

**Table 2:** Demographic characteristics of participants (*N* = 691).

Group	*n* (%)
Gender	
Boys	339 (49.0)
Girls	352 (50.9)
School year	
5th grade	375 (54.2)
6th grade	316 (45.7)
Socioeconomic status	
Low-SES	233 (33.7)
Average-SES	268 (38.7)
High-SES	190 (27.5)
Geographic distribution	
Urban schools	368 (53.3)
Rural schools	323 (46.7)

### Procedure

Children were assessed collectively in their regular classroom. For this purpose, initial contact was established with each school to present the aims and scope of the study and to obtain formal authorization from the school board. Upon approval, school administrators distributed an invitation letter to families, describing the purpose of the study and informing them that students would be assessed on tasks related to language and mathematics. An informed consent form was included, which required the signature of a parent or legal guardian to authorize participation. Ethical approval for the study was obtained from the Ethics Committee of the University of Málaga (614 CEUMA 16-2020-H), and all procedures were carried out in accordance with the Declaration of Helsinki.

The assessment protocol involved training speech therapists and teachers at each participant school. A briefing session was held to explain test contents, student instructions, and the overall purpose of the study to ensure consistent implementation of the assessment procedures across all participating schools. Tablets were used to administer the digital screening battery. Before each session, students were registered on the digital platform and assigned to a test group. Assessments were conducted in groups of 20 students during regular school hours. The full process, including login, instructions and testing lasted approximately 1 hour.

### Instruments

The exceptional circumstances imposed by the COVID-19 pandemic necessitated an innovative approach to ensure both the feasibility and quality of data collection. Due to health-related restrictions, external evaluators were not granted access to most schools. In response, a self-administered digital assessment protocol was specifically developed for this study. The protocol was implemented through an internet-based platform designed by our research group, which was optimized for tablet accessibility. This platform enabled the collective and computerized administration of all tasks in compliance with health and logistical constraints.

### Reading measures

Given the absence of in-person supervision, traditional oral reading tests—particularly those requiring word and pseudoword decoding—were replaced with two lexical decision tasks: one favoring word recognition and the other emphasizing pseudoword processing.

#### Lexical decision task

Participants were instructed to indicate whether the letter string presented on the screen was a real word or a pseudoword. Responses were given by pressing “YES” if the stimulus was judged to be a real word, and “NO” if it was considered a pseudoword. The task was divided into two separate tests: one biased toward words and the other biased toward pseudowords. Each test consisted of three subtests organized by word length: bisyllabic, trisyllabic, and quadrisyllabic items. In the word-biased lexical decision test, each subtest included 45 stimuli, of which 36 were real words and 9 were pseudowords, resulting in a total of 135 stimuli. In the pseudoword-biased lexical decision test, each subtest also included 45 stimuli, but with 36 pseudowords and 9 real words, for a total of 135 stimuli. In both tasks, the number of correct responses per minute was recorded. Each lexical decision test consisted of 135 items in total.

#### Reading fluency

Adapted from the Reading Fluency subtest of the Woodcock-Muñoz Battery III, this task assessed the ability to comprehend short, simple sentences under time pressure (Muñoz-Sandoval et al., 2005). Participants read each sentence silently and determined whether it was semantically true or false (e.g., “My mother eats busses”). They responded by selecting “YES” or “NO.” The task was timed (3 min), and the final score was computed as the number of correct responses minus the number of errors. Maximum score: 115. Reported reliability of the original version is 0.90 (Muñoz-Sandoval et al., 2005).

### Mathematics measures

#### Symbolic magnitude comparison

This task evaluated the participant’s ability to compare symbolic numerical magnitudes. Pairs of numbers were presented simultaneously, and participants selected the numerically larger value. The items increased in difficulty from single-digit (items 1–10), to two-digit (11–36), and three-digit comparisons (37–62). Both numerical distance (close vs. far) and compatibility effects were manipulated (Landerl and Kölle, 2009). Compatible trials featured numbers with both larger tens and units digits (e.g., 32–85), while incompatible trials contained conflicting magnitudes (e.g., 51–27). The task was limited to 1 min. Maximum score: 62 correct responses. Cronbach’s alpha = 0.95.

#### Arithmetic task

This task assessed mental calculation abilities across three levels of difficulty: single-digit (10 items), two-digit (12 items), and three-digit (12 items) addition and subtraction problems. Participants judged whether the presented solution was correct or incorrect. The total number of correct responses completed within 1 min constituted the final score. Maximum score: 34. Cronbach’s alpha = 0.78.

### Classification criteria

Prevalence estimates were based on participants’ performance in the screening tasks. Raw scores of each task were converted into z-score within grade group, and a cut-off of −1 standard deviation (SD;16th percentile) was applied to define risk status. Risk groups were defined as follows: (1) The RDyx group comprised students who scored at or below the 1 SD in both reading measures: lexical decision task (either the word-biased or the pseudoword-biased test) and reading fluency task. (2) The RDC group included students who scored at or below the −1 SD in both mathematics measures: symbolic magnitude comparison and arithmetic. (3) The comorbid RDyx + RDC group consisted of students who simultaneously met the criteria for both RDyx and RDC. (4) Finally, the NA group comprised students who scored above the – 1 SD in both domains.

The 1 SD (16th percentile) cut-off was chosen in line with previous epidemiological studies on learning difficulties (e.g., Lewis et al., 1994; Moll et al., 2014) and has been applied to identify children at risk for dyslexia and dyscalculia (Martin and Fuchs, 2022). This threshold reflects performance one standard deviation below the mean, indicating marked underachievement while being less stringent than clinical diagnostic criteria (typically 1.5 or 2 SD) (Moeller et al., 2012; Poulsen et al., 2023). Consistent with the aims of the present study, which focused on identifying risk rather than establishing a clinical diagnosis, the 1 SD cut-off was applied uniformly across all tasks to ensure comparability and consistency in classification.

### Statistical plan

All analyses were conducted in R (version 4.4.3). Prevalence estimates for each LDs risk group (RDyx, RDC and RDyx + RDC) were calculated with 95% confidence intervals (CIs). Differences across categorical variables (sex, SES) were examined using chi-square tests (χ^2^), with Cramér’s V reported as a measure of effect size. Logistic regression models were additionally estimated to provide odds ratios (ORs) with 95% CIs, thereby complementing the descriptive analyses and accounting for potential confounding effects. Exploratory and confirmatory factor analyses were conducted to examine the construct validity of the reading and mathematics tasks used for classification. A post-hoc power analysis was also carried out to assess the adequacy of the sample size.

## Results

An exploratory factor analysis (EFA) was first conducted to examine the latent structure of the tasks. Sampling adequacy was acceptable (KMO = 0.75), and Bartlett’s test of sphericity confirmed sufficient inter-item correlations, *χ*^2^(10) = 982.50, *p* < 0.001. The EFA indicated that a two-factor solution provided a better account of the data than a unidimensional model. While the one-factor model explained 42% of the variance and showed limited fit (RMSEA = 0.097), the two-factor model accounted for 59% of the variance and exhibited excellent fit, *χ*^2^(1) = 1.27, *p* = 0.26, RMSEA = 0.02, TLI = 0.997, CFI ≈ 1.

A confirmatory factor analysis (CFA) was subsequently performed to test this bifactorial structure, specifying two correlated latent factors: Reading (Lexical Decision: Words, Lexical Decision: Pseudowords, Reading Fluency) and Mathematics (Symbolic Magnitude Comparison, Arithmetic Task). The model demonstrated good fit, χ^2^(4) = 17.45, *p* = 0.002, CFI = 0.986, TLI = 0.966, RMSEA = 0.07, 90% CI [0.038–0.105], SRMR = 0.026. Standardized loadings ranged from 0.65 to 0.85 for the reading tasks and from 0.44 to 0.61 for the mathematics tasks. The correlation between the two latent factors was moderate to high (r = 0.66, p < 0.001), indicating that although reading and mathematics represent distinct constructs, they share substantial common variance.

### Prevalence rate

In the total sample (N = 691), the prevalence rates were 8.5% (95% CI [6.6–10.9]) for RDyx, 4.2% (95% CI [2.8–6.0]) for RDC, and 2.0% (95% CI [1.1–3.4]) for the comorbid group (RDyx + RDC) (see Table 3). An asymmetrical pattern of comorbidity was also observed: among children identified with RDC, 48.3% (95% CI [29.4–67.5]) also exhibited reading difficulties. In contrast, only 23.7% (95% CI [13.6–36.6]) of those classified with RDyx showed mathematical difficulties.

**Table 3:** Prevalence of participants classified by learning disorder risk group and gender.

Group (*N*)	RDyx		RDC		RDyx+RDC	
	*n* (%)	95% CI	*n* (%)	95% CI	*n* (%)	95% CI
Total sample (691)	59 (8.5)	6.6–10.9	29 (4.2)	2.8–6.0	14 (2.0)	1.1–3.4
Gender						
Girl (352)	36 (10.2)	7.3–13.9	22 (6.3)	4.0–9.3	11 (3.1)	1.6–5.5
Boy (339)	23 (6.8)	4.3–10.0	7 (2.1)	0.8–4.2	3 (0.9)	0.2–2.6

RDyx, risk of dyslexia; RDC, risk of dyscalculia; RDyx + RDC, comorbid dyslexia-dyscalculia risk. Numbers, percentages (in brackets), and 95% CI, confidence interval are reported. Percentages for the total sample are calculated with the whole sample (*N* = 691). Percentages for boys and girls are calculated within each subgroup (boys *N* = 339; girls *N* = 352).

To determine whether the observed co-occurrence of reading and mathematics difficulties exceeded what would be expected under statistical independence, the expected prevalence was calculated by multiplying the base rates of RDyx and RDC: (59/691) × (29/691) × 100 ≈ 0.36%. A chi-square test confirmed that the observed comorbidity rate (2.0%) was significantly higher than expected by chance, *χ*^2^(1, *N* = 691) = 61.20, *p* < 0.001, Cramér’s V = 0.29. These findings are consistent with previous research (e.g., Dirks et al., 2008; Moll et al., 2014), which also reported comorbidity rates exceeding those predicted under models of statistical independence.

### Gender differences

Gender ratios (boy:girl) were 0.64:1 for the RDyx group (23 boys, 36 girls), 0.32:1 for the RDC group (7 boys, 22 girls), and 0.27:1 for the comorbid RDyx + RDC group (3 boys, 11 girls), suggesting an overrepresentation of girls across all three categories. In the RDyx group, the prevalence was 10.2% for girls and 6.8% for boys; however, this difference was not statistically significant, *χ*^2^(1, *N* = 691) = 2.62, *p* = 0.10, Cramér’s V = 0.06 (see Table 3). Logistic regression confirmed the absence of a significant sex effect, OR = 1.56, 95% CI [0.91–2.73], *p* = 0.10. In contrast, a significant gender difference was observed in the RDC group, where prevalence was 6.3% for girls and 2.1% for boys, *χ*^2^(1, *N* = 691) = 7.52, *p* = 0.006, Cramér’s V = 0.10. Logistic regression indicated that girls had three times the odds of RDC compared to boys, OR = 3.16, 95% CI [1.39–8.09], *p* = 0.009. In the comorbid group (RDyx + RDC), prevalence was 3.1% for girls and 0.9% for boys, indicating a higher proportion of girls. The chi-square test confirmed a significant association, *χ*^2^(1, *N* = 691) = 4.36, *p* = 0.03, Cramér’s V = 0.07, and the odds of comorbidity were higher for girls OR = 3.60, 95% CI [0.94–20.31]. However, Fisher’s exact test yielded a marginal result (*p* = 0.05). A post-hoc power analysis indicated limited statistical power (≈59%); therefore, these findings should be interpreted with caution due to the small number of comorbid cases.

### Influence of socioeconomic status on prevalence of learning difficulties

To examine whether socioeconomic status (SES) influenced the prevalence of learning difficulties, a series of chi-square tests and logistic regressions were conducted for RDyx, RDC, and their comorbidity.

For RDyx, prevalence was highest in the low SES group (12.0%), followed by the high SES group (7.8%) and the average SES group (5.9%) (see Table 4). The chi-square test indicated a marginal association between SES and RDyx, *χ*^2^(2, *N* = 691) = 5.97, *p* = 0.05, Cramér’s V = 0.09. However, logistic regression did not confirm a significant SES effect when comparing low versus high SES OR = 1.59, 95% CI [0.83–3.15], *p* = 0.16. For RDC, prevalence varied minimally across SES groups (3.4% in the low SES group, 5.6% in the average SES group, and 3.1% in the high SES group). The chi-square test indicated no significant association between SES and RDC, *χ*^2^(2, *N* = 691) = 2.15, *p* = 0.34, Cramér’s V = 0.05. Logistic regression likewise revealed no significant SES effect when comparing low versus high SES OR = 1.09, 95% CI [0.37–3.36], *p* = 0.87. Finally, comorbidity rates were relatively stable across SES levels, with prevalence estimates of 2.1% in the low SES group, 2.2% in the average SES group, and 1.5% in the high SES group. The chi-square test confirmed the lack of an association between SES and comorbidity, *χ*^2^(2, *N* = 691) = 0.26, *p* = 0.87, Cramér’s V = 0.01. Consistently, no significant differences were detected when comparing low versus high SES (OR = 1.37, 95% CI [0.26–8.91], *p* = 0.74; Fisher’s exact test). A post-hoc power analysis indicated extremely limited statistical power (≈7%), underscoring the need for cautious interpretation given the small number of comorbid cases.

**Table 4:** Prevalence of participants classified by learning disorder risk group and SES.

SES (*N*)	RDyx		RDC		RDyx+RDC	
	*n* (%)	95% CI	*n* (%)	95% CI	*n* (%)	95% CI
Low (233)	28 (12.0)	8.1–16.9	8 (3.4)	1.5–6.7	5 (2.1)	0.7–4.9
Average (268)	16 (5.9)	3.5–9.5	15 (5.6)	3.2–9.1	6 (2.2)	0.8–4.8
High (190)	15 (7.8)	4.5–12.7	6 (3.1)	1.2–6.7	3 (1.5)	0.3–4.5

RDyx, risk of dyslexia; RDC, risk of dyscalculia; RDyx + RDC, comorbid dyslexia-dyscalculia risk; SES, socioeconomic status. Numbers, percentages (in brackets), and 95% CI, confidence interval are reported. Percentages are calculated relative to the total number of participants in each SES category.

## Discussion

### Prevalence patterns and comorbidity

The present study first aimed to estimate the prevalence of RDyx, RDC, and their comorbidity in a large sample of Spanish primary school students, and to analyze how these learning disorders vary across gender and socioeconomic status (SES). Results indicate that 8.5% of students met the criteria for RDyx. This prevalence rate aligns with estimates reported in studies conducted within Spanish-speaking populations, which range from 3.2 to 10.9% (e.g., Carrillo et al., 2011; Cubilla-Bonnetier, 2024; Jiménez et al., 2009). In turn, the 4.2% prevalence of RDC is consistent with previous international and Spanish estimates, which generally range from 3 to 7% (e.g., Barbaresi et al., 2005; Devine et al., 2013; Luoni et al., 2023). These figures confirm that these disorders are frequent challenges in the Spanish school populations and support the viability of the testing procedure used here.

One of the most relevant contributions of this study lies in that this is the first study to report rates of comorbidity between RDyx and RDC in a large population-based Spanish sample. Although the prevalence falls within the range limits of previous studies pointing to the association of both disorders, the observed co-occurrence rate was a moderate 2% despite the lenient cut-off used and contrast with the high comorbidity rates found in affected samples (Landerl and Moll, 2010). This result may be due to that our participants came from an unselected sample and they had a narrow age range, indicating the importance of studying samples representative of the general population.

Interestingly, an asymmetrical pattern of comorbidity was identified: 48.3% of students with RDC presented concurrent reading difficulties, whereas only 23.7% of students with RDyx exhibited mathematical difficulties. Similar asymmetrical co-occurrence patterns have been reported in previous studies (Landerl and Moll, 2010; Koponen et al., 2018), reinforcing the notion that comorbidity is not bidirectional. This asymmetry may stem from fundamental differences in the cognitive bases that underlie reading and arithmetic difficulties. The overlap has been attributed to that many mathematical operations (e.g., counting, remembering facts or learning multiplication tables) relay on verbal skills (de Smedt et al., 2010; Hecht et al., 2001; Vanbinst et al., 2020). Then individuals with phonological deficits may be at risk of both dyslexia and arithmetic difficulties. However, arithmetic competence involves other specific components as number sense or quantity representation (Dehaene, 1997) that are not essential for acquiring reading skills. As a result, there may be more children with arithmetic difficulties who exhibit reading problems than dyslexics with concurrent difficulties in mathematics.

### Gender differences in learning disorders

In contrast with the reported male predominance in reading disorders in some studies (e.g., Rutter et al., 2004; Quinn and Wagner, 2015), the present findings align with previous population-based studies (Dirks et al., 2008; Jiménez et al., 2011; Landerl and Moll, 2010; Shaywitz et al., 1990), showing no significant difference between the rate of boys and girls with dyslexia. One possible explanatory factor is referral bias. Shaywitz et al. (1990) suggested that the overrepresentation of boys in school-identified samples may reflect the tendency of educators to refer children who exhibit more overt externalizing behaviors, such as hyperactivity or conduct problems. In contrast, when dyslexia is identified through objective and systematic evaluations, gender ratios tend to be more balanced. Supporting this view, Vogel (1990) notes that girls are often underrepresented in referred samples precisely because their behavioral profile is less disruptive. In the present study, RDyx was identified through a systematic assessment across the entire sample. This approach likely minimized biases and may explain the absence of significant gender differences in RDyx prevalence in our results.

With respect to the gender ratio in the RDC and comorbid groups, a preponderance of girls was observed in this study. The higher prevalence of girls in the RDC group is consistent with previous studies (Dirks et al., 2008; Moll et al., 2014; Poltz et al., 2025). Several studies suggest that this overrepresentation may reflect the impact of affective and sociocultural variables rather than a gender-based predisposition. Factors such as math anxiety, gender stereotypes, and low confidence in their own mathematical ability have been shown to negatively influence the performance of girls in this domain (Goetz et al., 2013; Hyde and Mertz, 2009; Rossi et al., 2022). These vulnerabilities appear to disproportionately affect girls and may contribute to the onset or exacerbation of learning difficulties in mathematics (Dowker et al., 2016). Moreover, classroom dynamics and differential teacher expectations may reinforce gendered patterns of achievement and self-perception, further amplifying the influence of sociocultural factors on academic outcomes (Else-Quest et al., 2010; Dowker et al., 2016). Further research is needed to determine whether the observed gender differences in mathematics reflect a genuine biological predisposition or are primarily driven by sociocultural influences.

### Socioeconomic disparities

One of the clearest trends observed in this study was the higher prevalence of reading difficulties among students from low SES backgrounds. Although the strength of this association was only marginally significant, it is in accordance with a robust body of research linking socioeconomic disadvantage to poorer literacy outcomes (Buckingham et al., 2014; Noble et al., 2015; Pan et al., 2005; Romeo et al., 2022). One plausible explanation is that lower SES constrains the early language experiences of children in both quantity and quality. As Hoff (2003) demonstrated, children from higher SES families are typically exposed to more linguistically rich interactions, characterized by a greater number of utterances, broader vocabulary, and more complex syntactic structures in maternal speech. These differences in language input contribute to foundational skills such as phonological awareness, vocabulary acquisition, and letter knowledge, which in turn facilitate reading development. In contrast, children from disadvantaged backgrounds may experience more limited verbal interaction, less frequent joint attention episodes, and reduced opportunities for scaffolded learning at home, all of which can hinder the acquisition of core literacy skills (Buckingham et al., 2014; Catts and Petscher, 2022; Hoff, 2003; Neuman et al., 2018).

Unexpectedly, no significant differences were observed in the distribution of RDC across SES groups, nor in the comorbid group. This finding suggests that SES may exert a stronger influence on reading skills than on mathematical performance. Although few epidemiological studies have specifically examined the role of SES in the prevalence of mathematics disability and its comorbidity with reading disability, existing evidence tends to show higher rates of mathematical difficulties among students from disadvantaged backgrounds (Gross-Tsur et al., 1996; Luoni et al., 2023). Nevertheless, the relationship between SES and mathematical outcomes may be more complex or potentially mediated by factors not directly assessed in the present study. Further investigation should examine the influence of SES on both disorders not only in isolation but also in their comorbid manifestations. This remains a growing area of interest, as environmental and contextual factors may exacerbate or mitigate the effects of genetic predispositions and shape the severity and manifestation of dyslexia and dyscalculia (Catts and Petscher, 2022; Girard et al., 2022; Macdonald and Deacon, 2019).

### Limitations and implications for practice

This study has several limitations that should be considered when interpreting the findings. First, although the sample was large, it was restricted to public schools in a single Spanish province, which may limit generalizability to other educational contexts such as private or semi-private schools. Second, the classification relied on a cutoff of −1 SD (16th percentile). While this criterion is commonly used in epidemiological studies, it does not equate to a clinical diagnosis and may either overestimate or underestimate prevalence depending on the chosen threshold. Third, the relatively small number of students identified in the comorbid group reduced statistical power for analyzing interaction effects.

Despite these limitations, the study provides valuable insights into the prevalence and sociodemographic distribution of risk for dyslexia and dyscalculia in Spanish primary education. Importantly, the screening approach developed here has the advantage of being low-cost, brief, and feasible for implementation in school settings. For practice, our findings underscore the need to move toward universal school-based screening protocols that can be administered by trained teachers or educational staff with minimal resources. Such tools may play a key role in ensuring that children at risk are identified early enough to benefit from targeted support.

A particularly relevant implication is the extension of this screening approach to earlier developmental stages. Administering similar protocols in the first years of primary school—or even at the end of preschool—would allow for the detection of risk before reading and mathematics difficulties consolidate. Early identification increases the likelihood that preventive interventions can be applied at a stage when they are most effective. Furthermore, incorporating additional measures, such as RAN tasks, phonological awareness, or broader language assessments, would provide a more comprehensive profile of children’s strengths and weaknesses and enhance the robustness of risk classification.

In summary, while this study demonstrates the feasibility of population-based digital screening at the end of primary school, future research should aim to broaden the age range and enrich the set of tasks used. By doing so, educational systems will be better equipped to detect risk trajectories earlier, to tailor interventions with greater precision, and ultimately to promote more equitable educational opportunities.

## Conclusions and practical implications

This study offers the first population-based estimates of the prevalence of risk for dyslexia, dyscalculia, and their comorbidity in Spanish primary education, highlighting their frequent overlap and the asymmetrical nature of comorbidity. The findings show that dyscalculia often co-occurs with reading difficulties to a greater extent than the reverse, supporting the view that learning disorders are better understood as multidimensional and interconnected rather than isolated conditions. Moreover, the observed differences by gender and socioeconomic status reveal that these risk factors are unevenly distributed across the population, emphasizing the need to consider sociodemographic influences when designing screening and support strategies.

The novelty of this work lies in combining large-scale prevalence estimates with the systematic analysis of sociodemographic correlates, thereby contributing unique evidence from Spain to the international literature. The adoption of brief, computerized screening tools demonstrates that it is possible to generate robust epidemiological data in school settings without relying on clinical referrals, an approach that aligns with current calls for universal and scalable identification methods.

Future research should extend this approach to younger cohorts and broaden the assessment battery to include additional markers of risk, enabling the identification of children before learning difficulties consolidate. Longitudinal studies will also be necessary to track developmental trajectories and to examine how early risk translates into persistent difficulties or response to intervention. By integrating epidemiological evidence with practical screening solutions, this study contributes to advancing both scientific understanding and educational practice, offering a foundation for more equitable and timely support for students at risk.

The results highlight the need for systematic, school-based screening to detect students at risk for dyslexia and dyscalculia before difficulties consolidate. Implementing short, digital tools in classrooms can provide educators with timely information to guide support, particularly for students from disadvantaged backgrounds who face higher vulnerability. Schools and policymakers should prioritize universal screening protocols, ensure teacher training for their administration, and allocate resources to early intervention programs. By embedding these practices into the educational system, risk can be addressed proactively, reducing long-term academic and social consequences.

## Data Availability

The datasets presented in this study can be found in online repositories. The names of the repository/repositories and accession number(s) can be found at: Open Science Framework (OSF) repository: https://osf.io/nx582/.
